# Supplementary material for: Room-temperature valence transition in a strain-tuned perovskite oxide
Source: Nat Commun. 2022 Dec 15;13:7774. doi: 10.1038/s41467-022-35024-8 (PMC9755214; doi:10.1038/s41467-022-35024-8)
Supplement: Supplementary file 1 — Supplementary Information [file 41467_2022_35024_MOESM1_ESM.pdf]

Supplementary Information for

Room-Temperature Valence Transition in a Strain-Tuned Perovskite Oxide

Vipul Chaturvedi[1], Supriya Ghosh[1], Dominique Gautreau[1,2], William M. Postiglione[1], John E. Dewey[1], Paige E. Quarterman[3], Purnima P. Balakrishnan[3], Brian J. Kirby[3], Hua Zhou[4], Huikai Cheng[5], Amanda Huon[6], Timothy Charlton[6], Michael R. Fitzsimmons[6,7], Caroline Korostynski[1], Andrew Jacobson[1], Lucca Figari[1], Javier Garcia Barriocanal[8], Turan Birol[1], K. Andre Mkhoyan[1], and Chris Leighton[1]*

[1]*Department of Chemical Engineering and Materials Science, University of Minnesota, Minneapolis, Minnesota 55455, USA*

[2]*School of Physics and Astronomy, University of Minnesota, Minneapolis, Minnesota 55455, USA*

[3]*NIST Center for Neutron Research, National Institute of Standards and Technology, Gaithersburg, Maryland 60439, USA*

[4]*Advanced Photon Source, Argonne National Laboratory, Lemont, Illinois 60439, USA*

[5]*Thermo Fisher Scientific, Hillsboro, Oregon 97124, USA*

[6]*Neutron Scattering Division, Oak Ridge National Lab, Oak Ridge, Tennessee 37830, USA*

[7]*Department of Physics and Astronomy, University of Tennessee, Knoxville, Tennessee 37996, USA*

[8]*Characterization Facility, University of Minnesota, Minneapolis, Minnesota 55455, USA*

*email: leighton@umn.edu

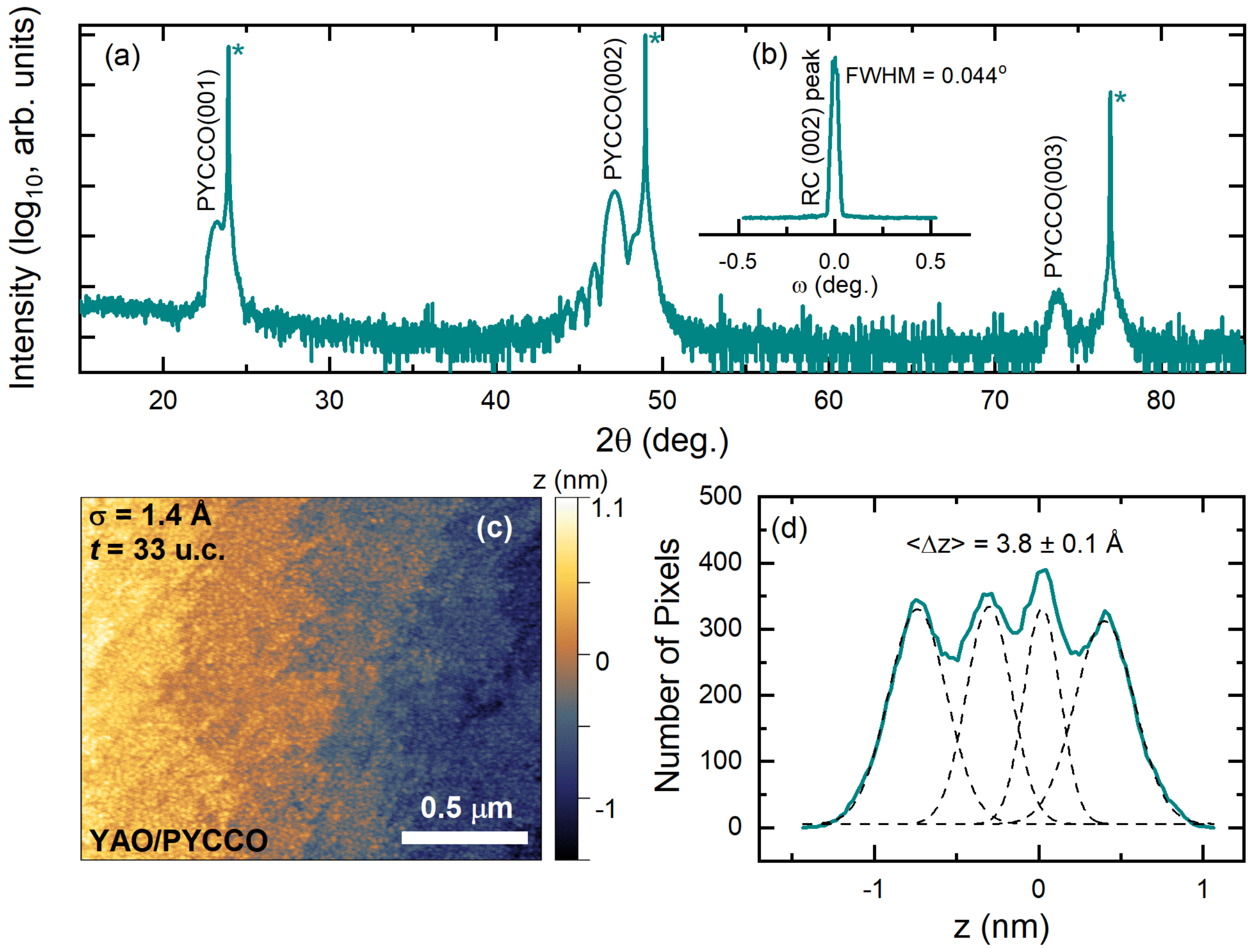

Figure S1: Additional X-ray and atomic force microscopy characterization. Wide-range specular X-ray diffraction scan (a) and 002 rocking curve (RC) (b) from a 31-unit-cell-thick $(Pr_{0.85}Y_{0.15})_{0.7}Ca_{0.3}CoO_{3-\delta}$ film on YAO(101). These are lab X-ray data taken using Cu K_{α} radiation. Substrate reflections are labelled with an "*", and the full-width at half-maximum (FWHM) of the RC is shown. As noted in the main text, single-phase epitaxial films are evidenced. (c) Contact-mode atomic force microscopy height image of a 33-unit-cell-thick $(Pr_{0.85}Y_{0.15})_{0.7}Ca_{0.3}CoO_{3-\delta}$ film on YAO(101), leveled to the visible terraces; the root-mean-square roughness from the displayed region is 1.4 Å. (d) Number of pixels *vs.* height (z) histogram from the image in (c). The shown multi-peak Gaussian fit yields an average step height $<\Delta z>$ = 3.8 ± 0.1 Å (one standard deviation of random fitting error), *i.e.*, very close to one unit cell, as mentioned in the main text.

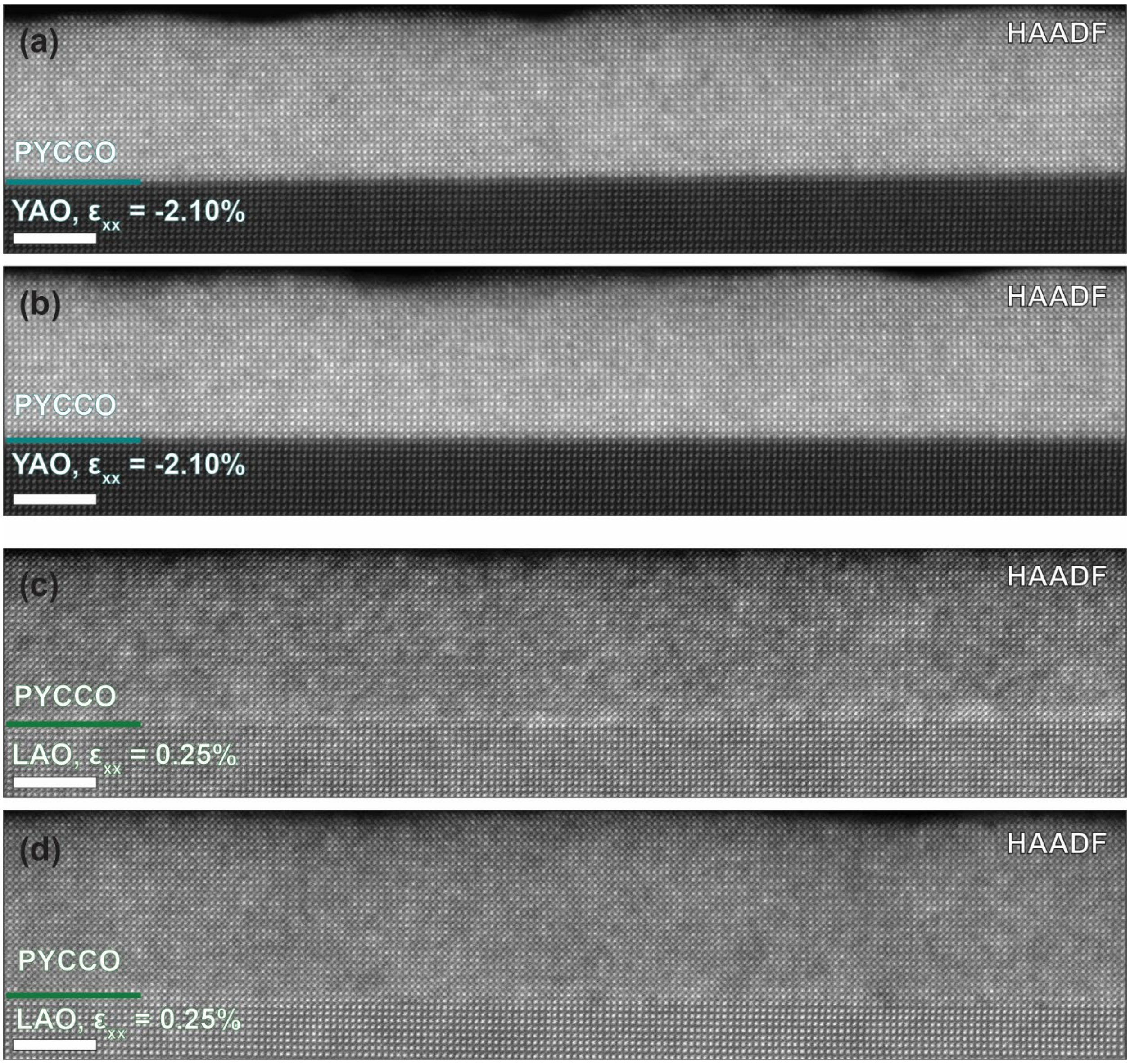

Figure S2: Additional electron microscopy characterization. Atomic-resolution high-angle annular-dark-field scanning transmission electron microscopy (HAADF-STEM) images of ~26-unit-cell-thick $(Pr_{0.85}Y_{0.15})_{0.7}Ca_{0.3}CoO_{3-\delta}$ (PYCCO) films on YAO(101) (ε_{xx} = -2.10 %) and LAO(001) (ε_{xx} = 0.25 %) substrates. (a,b) HAADF-STEM images of different regions of a PYCCO film on YAO. (c,d) HAADF-STEM images of different regions of a PYCCO film on LAO. The scale bars are 5 nm, and the horizontal lines mark the interfaces. As noted in the main text, the local HAADF intensity variations due to doping fluctuations are evident throughout the images. No superstructure due to, *e.g*., O vacancy order, is observed in any case.

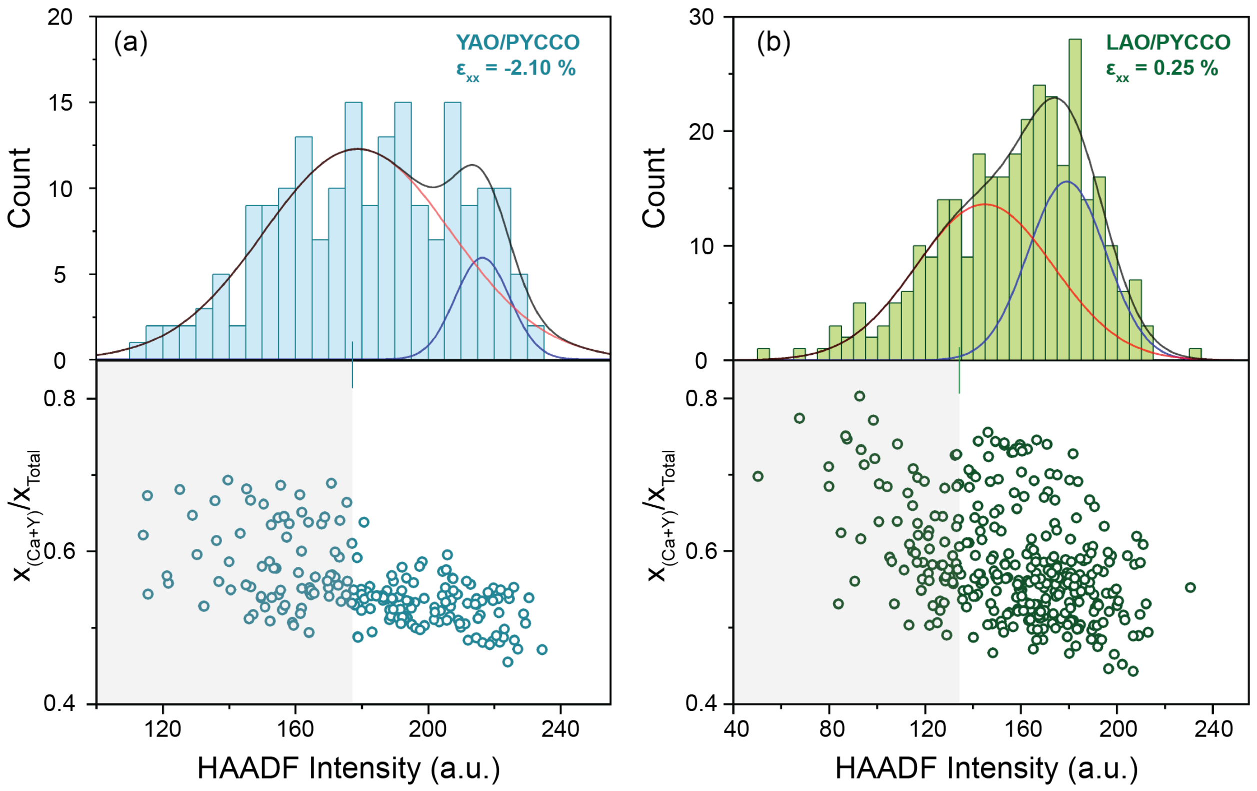

Figure S3: Statistical correlation between HAADF-STEM intensity variations and local substituent (Ca and Y) concentrations. (a,b) Normalized HAADF intensity distribution obtained from many atomic columns of PYCCO films (top panels). These distributions are superpositions of two Gaussians: a broader distribution for the lower intensity columns (red curves) and a narrower distribution for the higher intensity columns (blue curves). The black curves are the sums of the two. The bottom panels show the effective substituent (Ca and Y) concentration in the corresponding atomic columns. The $x_{(Ca+Y)}/x_{Total}$ is calculated from the intensities of the respective elements in the atomic-resolution STEM-EDX maps of Pr, Ca, and Y. At lower HAADF intensities (shaded regions), the substituent ratio is higher. Since $Z_{Ca} = 20$ and $Z_Y = 39$ are lower than $Z_{Pr} = 59$, substitution of Ca (and Y) will lower the effective-Z of a given atomic column, and hence lower the HAADF intensity as captured in these plots. Most notably, under tensile strain (on LAO, right panels) the two contributions to the HAADF intensity distribution are more distinct (top panel) and the local compositional fluctuations are stronger (bottom panel), as discussed in the main text.

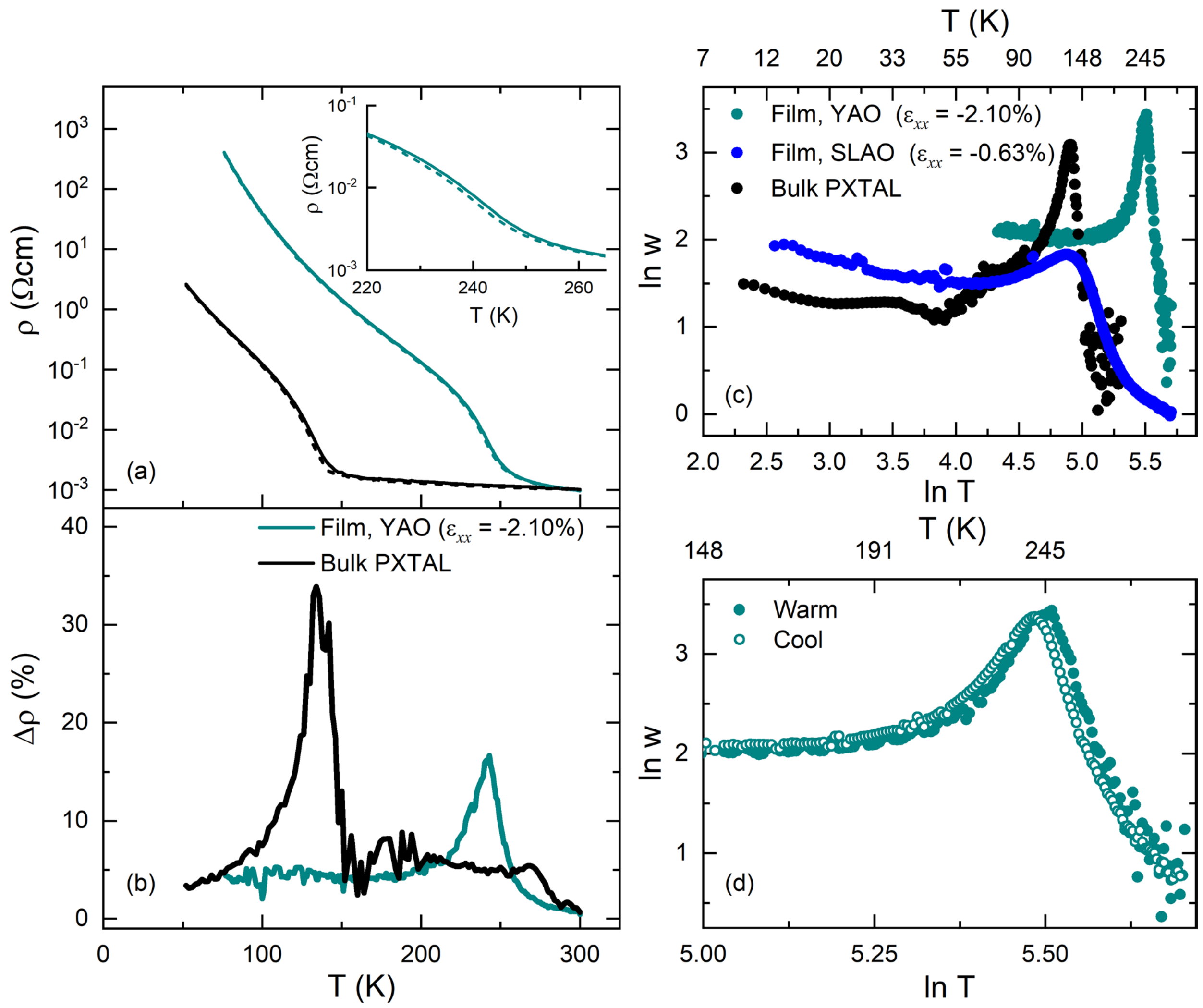

Figure S4: Transport analysis of hysteresis and transition width. (a) Resistivity *vs*. temperature ($\rho(T)$) ($\log_{10}$ scale) for a 28-unit-cell-thick $(Pr_{0.85}Y_{0.15})_{0.7}Ca_{0.3}CoO_{3-\delta}$ film on YAO(101) and a polycrystalline bulk (bulk PXTAL) sample of the same composition. Both warming (solid) and cooling (dashed) curves are shown (taken at 1 K/min). The inset in (a) shows a close-up near the transition temperature for the film on YAO. (b) T dependence of the thermal hysteresis, defined as $\Delta\rho(\%) = [(\rho_{\text{warming}} - \rho_{\text{cooling}})/\rho_{\text{cooling}}] \times 100\%$, from the data in (a). (c) "Zabrodskii plot" of ln w *vs*. ln T where $w = -d(\ln\rho)/d(\ln T)$, for the same data as in (a), along with a 33-unit-cell-thick film on SLAO(001). The use of a logarithmic derivative in such plots highlights the position and shape of the insulator-metal transition. On YAO, the elevated-temperature transition is seen to be as sharp as bulk, as noted in the main text, whereas on SLAO the transition is heavily broadened. (d) Close-

up Zabrodskii plot for the 28-unit-cell-thick $(Pr_{0.85}Y_{0.15})_{0.7}Ca_{0.3}CoO_{3-\delta}$ film on YAO(101) in (a-c), taken both during warming (solid) and cooling (open).

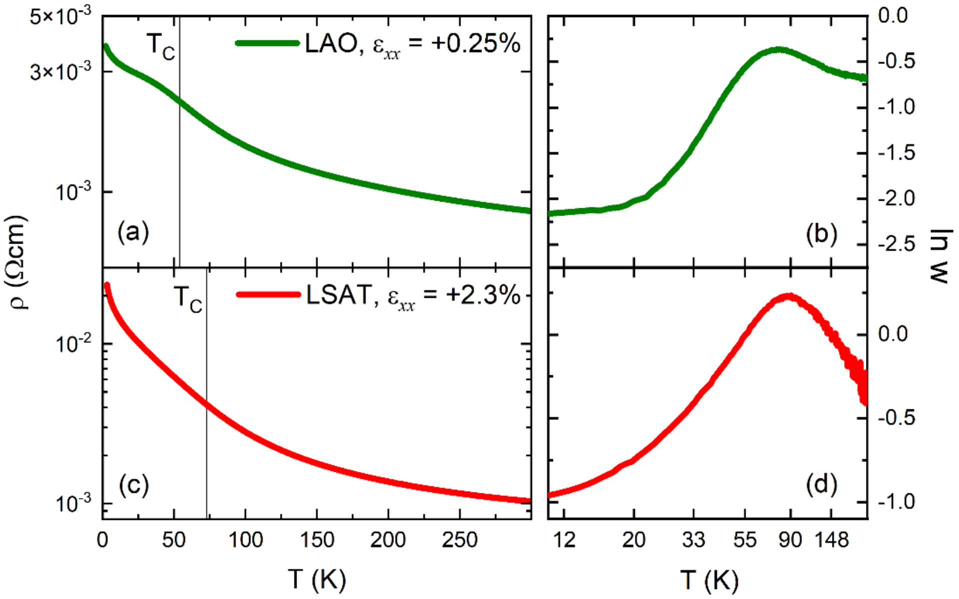

Figure S5: Additional transport analysis under tensile strain. Resistivity *vs.* temperature ($\rho(T)$) (left panels, $\log_{10}$ scale) and "Zabrodskii plots" (right panels, *i.e.*, ln w *vs.* T (log scale) where $w = -d(\ln\rho)/d(\ln T)$) for ~30-unit-cell-thick $(Pr_{0.85}Y_{0.15})_{0.7}Ca_{0.3}CoO_{3-\delta}$ films on (a,b) LAO(001) and (c,d) LSAT(001) substrates. Vertical solid lines denote the Curie temperature (T_C). Note the small but noticeable anomalies near T_C in (a) and (c), which appear as clear peaks in (b) and (d). Films on SLGO (1.67% tensile strain) were found to produce higher T_C of ~85 K, but with anomalously high resistivity associated with high surface roughness of this substrate.

Table S1: Summary of parameters extracted from refinement of polarized neutron reflectometry data on 56-unit-cell-thick $(Pr_{0.85}Y_{0.15})_{0.7}Ca_{0.3}CoO_{3-\delta}$(PYCCO) on LSAT (as shown in Fig. 4). The parameters are (from top to bottom) the nuclear scattering length density (ρ_{Nuc}), thickness (t), roughness (σ) (*i.e.*, Gaussian interface width), and magnetization (M) for each layer. Best fits were obtained with magnetically-dead top (*i.e.*, surface) and bottom (*i.e.*, interfacial) layers. These layers have zero magnetization but identical ρ_{Nuc} to the bulk (middle) layer. Note that PNR and, *e.g.*, atomic force microscopy, do not probe equivalent lateral areas and roughnesses, and are thus not exactly comparable. Unless otherwise noted, model parameter uncertainties represent ±2 standard deviations.

Sample	Substrate	Bottom PYCCO	Middle PYCCO	Top PYCCO
LSAT/PYCCO	ρ_{Nuc} = 5.16×10^{-6} Å^{-2}	ρ_{Nuc} = 4.55×10^{-6} Å^{-2}	ρ_{Nuc} = 4.55×10^{-6} Å^{-2}	ρ_{Nuc} = 4.55×10^{-6} Å^{-2}
	$t = \infty$	t = 4.85 Å ± 1.6 Å	t = 195.21 Å ± 0.6 Å	t = 9.97 Å ± 3.85 Å
	σ = 18.5 Å ± 1.2 Å	-	σ = 14.79 Å ± 0.14Å	-
	M = 0	M = 0	M = 0.90±0.01 μ_B/Co	M = 0

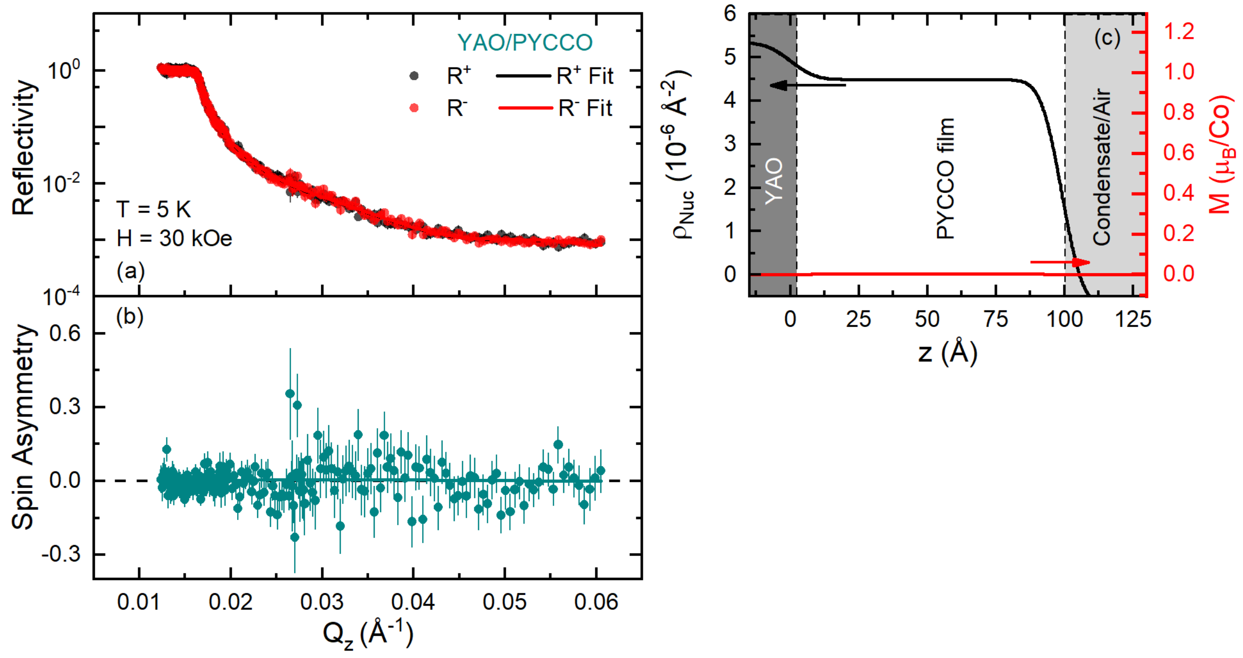

Figure S6: Polarized neutron reflectometry under compressive strain on $YAlO_3$. (a) Neutron reflectivity (R) *vs.* scattering wave vector (Q_z) from a 28-unit-cell-thick $(Pr_{0.85}Y_{0.15})_{0.7}Ca_{0.3}CoO_{3-\delta}$(PYCCO) film on YAO(101) at 5 K, in a 30-kOe (3-T) in-plane magnetic field (H). Black and red points denote the non-spin-flip channels R^+ and R^-, respectively, and the solid lines are the fits. (b) Spin asymmetry [SA = $(R^+-R^-)/(R^++R^-)$] *vs.* Q_z extracted from (a). (The data in (b) are raw, while the equivalent in Fig. 4(b) are averaged to a point spacing of 0.0005 $Å^{-1}$). Note that these PNR data, unlike those in Fig. 4, where taken in a half polarization configuration on the magnetism reflectometer at the Spallation Neutron Source, Oak Ridge National Laboratory. However, the magnetic field applied here is sufficient to isolate any spin-flip scattering from the non-spin-flip channels, as documented, for example, in Ref. [1]. (c) Depth (z) profiles of the nuclear scattering length density (ρ_{Nuc}, left-axis) and magnetization (M, right-axis) extracted from the fits to the YAO(101)/PYCCO data shown in (a,b). A low density overlayer on the film was required to fit the data, due to, *e.g.*, H_2O or hydrocarbon condensation. Unless otherwise noted, all uncertainties and error bars represent ±1 standard deviation. As discussed in the main text, the upper bound on the magnetization in the film is <0.01 μ_B/Co.

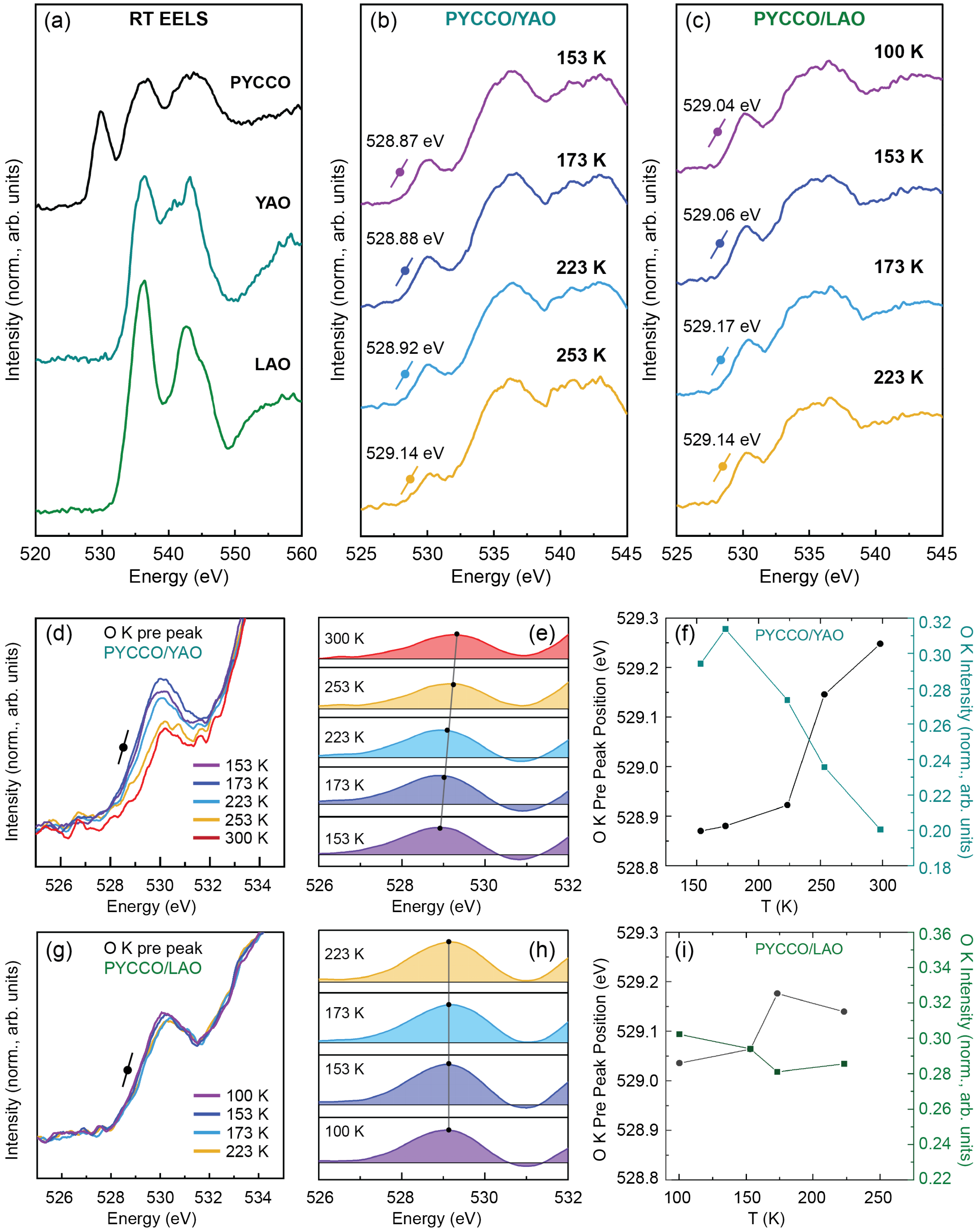

Figure S7: Additional EELS data from STEM/EELS (scanning transmission electron microscopy/electron energy loss spectroscopy) studies. (a) EELS O *K*-edge of a

$(Pr_{0.85}Y_{0.15})_{0.7}Ca_{0.3}CoO_{3-\delta}$(PYCCO) film, compared to a YAO and LAO substrate at room temperature (RT). The RT O *K*-edge data from the substrates were used for energy alignment of the temperature-dependent EELS data; EELS spectra were also normalized to the post-edge intensity (580-585 eV) to facilitate direct comparison. (b) Temperature (*T*)-dependent O *K*-edge data for ~30-unit-cell-thick PYCCO on YAO, showing a shift in the pre-peak position (as in Fig. 5(b)) under compressive strain, as more easily visualized in (d). (c) Equivalent *T*-dependent O *K*-edge data for ~30-unit-cell-thick PYCCO on LAO. As discussed below, the solid point and lines parallel to the pre-peak edges in (b) and (c) are used to track the position of the pre-peak *vs*. *T*. (d) Overlaid *T*-dependent (153 K to 300 K) EELS spectra near the O *K* edge pre-peak region from the ~30-unit-cell-thick PYCCO film on YAO shown in (b). Clear changes in fine structure are evident, as discussed below, and in the main text in connection with Fig. 5. (e) First derivative of the spectra in (d), obtained using 30-pixel averaging (a 1.5 eV energy range) to reduce noise. The peaks (marked) are thus the inflection points of the pre-peaks, which we use to track *T*-driven shifts in pre-peak position. The solid line connecting the peaks in (e) emphasizes the shift to lower energy as *T* is lowered. In (d), the black line illustrates the low-*T* slope while the black point marks the low-*T* inflection point. (f) Resulting YAO/PYCCO pre-peak position (left axis) and normalized peak intensity (right axis, integrated over 527-532 eV) *vs*. *T*. The pre-peak position from the analysis in (e) shifts by 0.37 eV from 153 K to 300 K. (g) Overlaid *T*-dependent (100 K to 223 K) EELS spectra near the O *K* edge pre-peak region from the ~30-unit-cell-thick PYCCO film on LAO shown in (c). Note the strikingly weak *T* dependence compared to (d). (h) First derivative of the spectra in (g), obtained using 30-pixel averaging (a 1.5 eV energy range) to reduce noise. The peaks (marked) are thus the inflection points of the pre-peaks, which we use to track *T*-driven shifts in pre-peak position. The solid line connecting the peaks in (h) emphasizes the lack of *T* dependence compared to (e). (i) Resulting LAO/PYCCO pre-peak position (left axis) and normalized peak intensity (right axis, integrated over 527-532 eV) *vs*. *T*. Note the weak *T* dependence compared to (f).

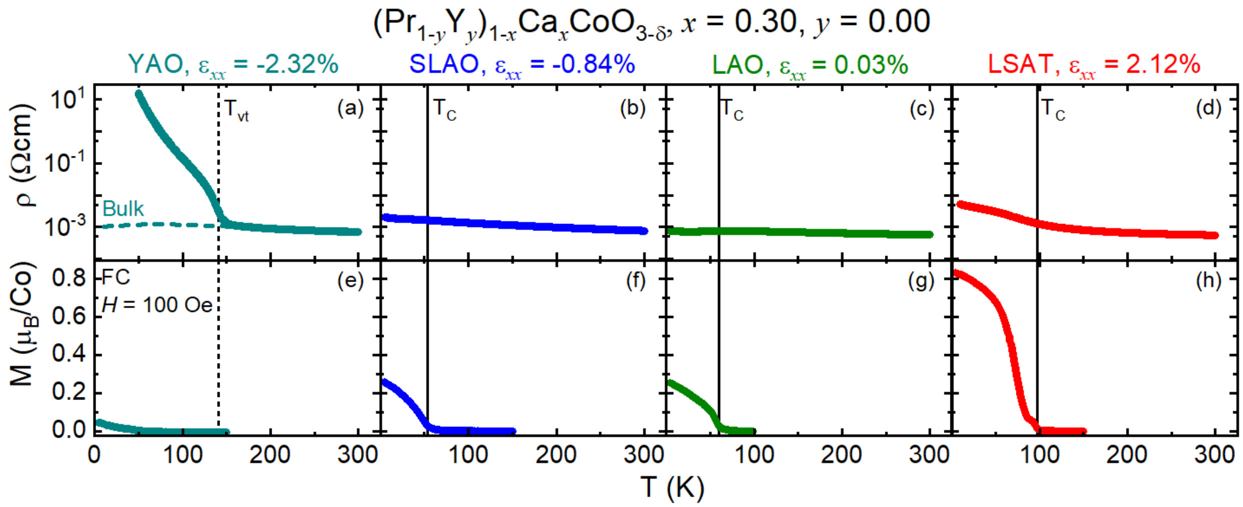

Figure S8: Transport and magnetometry data on strained $Pr_{0.7}Ca_{0.3}CoO_{3-\delta}$. Temperature ($T$) dependence of the resistivity (ρ) (top panels, $\log_{10}$ scale, taken on warming) and magnetization (M) (bottom panels) of 28-unit-cell-thick $Pr_{0.7}Ca_{0.3}CoO_{3-\delta}$ films (*i.e.*, x = 0.30, y = 0.00) on (a,e) YAO(101), (b,f) SLAO(001), (c,g) LAO(001), and (d,h) LSAT(001) substrates. The "in-plane strains" ε_{xx} are shown. M was measured in-plane in a 100 Oe (10 mT) applied field (H) after field-cooling in 10 kOe (1 T). Dashed and solid lines mark the valence transition temperature (T_{vt}) and Curie temperature (T_C). The T_C = 97 K in (h) is the largest achieved in this work.

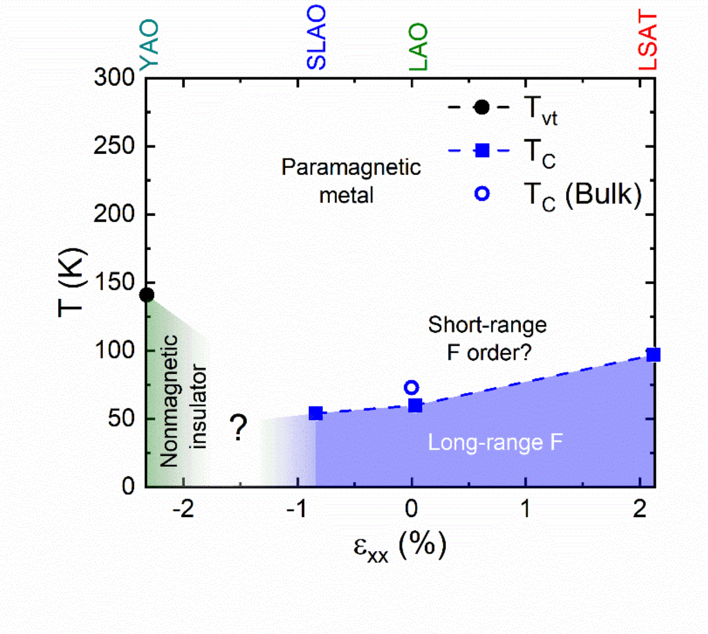

Figure S9: Temperature (*T*) *vs.* "in-plane strain" (ε_{xx}) "phase diagram" for $Pr_{0.7}Ca_{0.3}CoO_{3-\delta}$ (*i.e.*, PYCCO with *x* = 0.3, *y* = 0). Thin film (solid points) and bulk (open point) data are shown, with the relevant substrates indicated at the top. The valence transition temperature T_{vt} (circles) and Curie temperature T_C (squares) are plotted. Green, white, and blue phase fields indicate "nonmagnetic insulator", "paramagnetic metal", and "long-range ferromagnet (FM)", respectively.

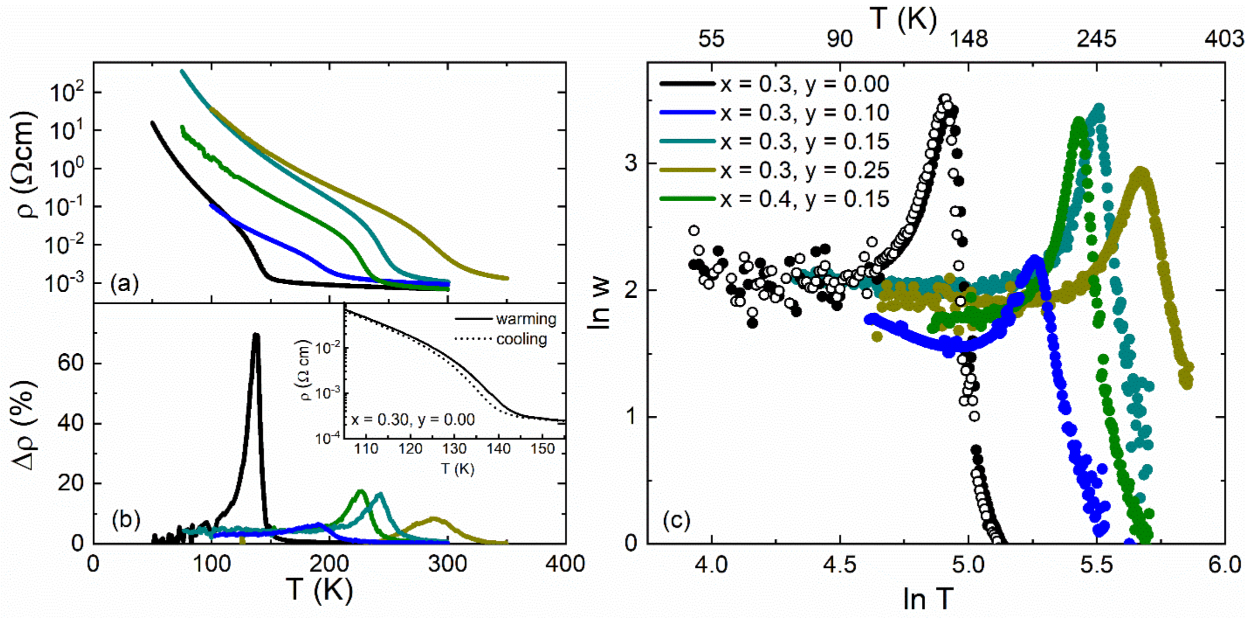

Figure S10: Additional transport analysis at multiple *x* and *y* in $(Pr_{1-y}Y_y)_{1-x}Ca_xCoO_{3-\delta}$ on $YAlO_3$. (a) Temperature (T) dependence of the resistivity (ρ) ($\log_{10}$ scale) for 28- to 38-unit-cell-thick $(Pr_{1-y}Y_y)_{1-x}Ca_xCoO_{3-\delta}$ films on YAO(101) for various x and y compositions (see legend in (c)). (b) T dependence of the thermal hysteresis, defined as $\Delta\rho(\%) = [(\rho_{warming} - \rho_{cooling})/\rho_{cooling}] \times 100\%$, measured at 1 K/min, for the films in (a). The inset in (b) shows a close-up of $\rho(T)$ near the transition for the x = 0.30, y = 0 case, for both cooling (dashed) and warming (solid). (c) "Zabrodskii plot" of ln w *vs.* ln T where $w = -d(\ln\rho)/d(\ln T)$, for the same data as in (a). Such plots highlight the position and shape of the insulator-metal transition. Note that the transition for $y = 0$ (black) is sharpest (a,b,c) and exhibits the strongest thermal hysteresis (b), as noted in the main text. Solid and open symbols denote warming and cooling, respectively.

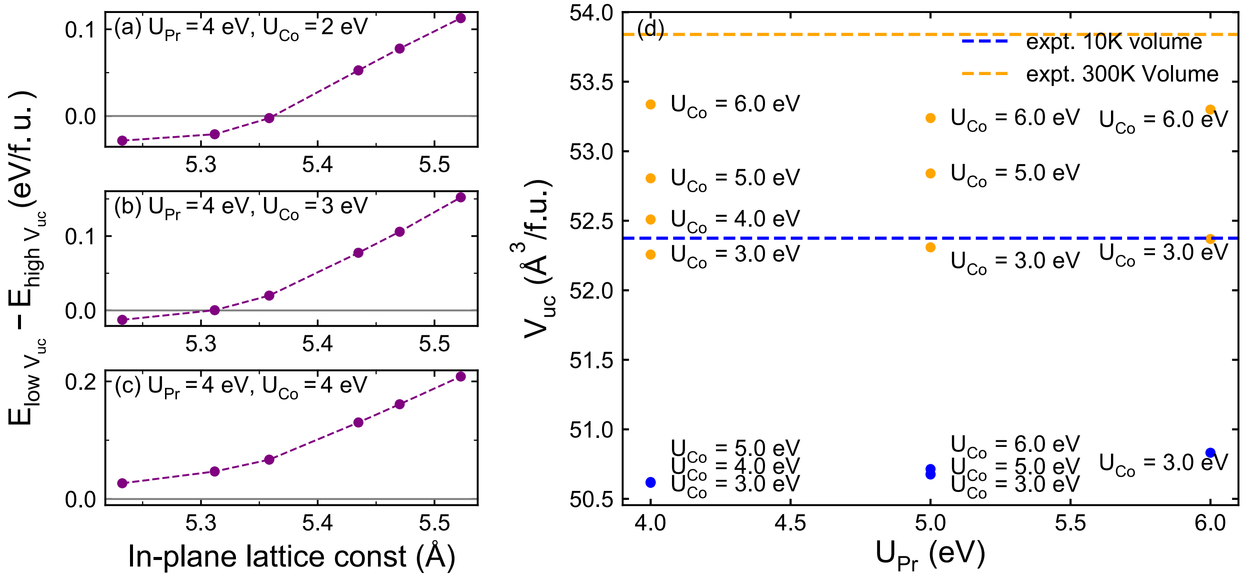

Figure S11: Supporting data for density functional theory calculations. (a-c) DFT+U-calculated energy differences between low-volume insulating and high-volume metallic states in $Pr_{0.5}Ca_{0.5}CoO_3$ (PCCO) as a function of the in-plane lattice constant (and thus biaxial strain), for different sets of U on Pr and Co. Since all reasonable U_{Pr} and U_{Co} values were found to underestimate the experimental volumes of both the high- and low-volume states, in the main text we report results using U values such that the DFT-predicted *difference* in bulk volumes is similar to that seen in experiment (U_{Pr} = 4 eV and U_{Co} = 3 eV).* Although the precise location of the transition predicted by DFT (*i.e.*, the sign change in ($E_{\text{low Vuc}} - E_{\text{high Vuc}}$) is sensitive to the choice of U values (see panels a-c), and should thus not be taken as exact, we find that for a wide range of U_{Pr} and U_{Co} the high-volume state becomes more stable/favorable under tensile strain (large in-plane lattice constant). Panel (d) shows the results for DFT-calculated volumes of bulk PCCO, compared to experiment (horizontal dashed lines). The absolute volumes of both the low- and high-volume states are underestimated by DFT, as noted above. U_{Pr} values of 5 eV and higher, in combination with U_{Co} values higher than 3 eV, overestimate the volume difference between the low- and high-volume states in bulk PCCO.

*Because the volumes are underestimated, the in-plane lattice constant corresponding to zero biaxial strain is smaller in our calculations than in experiment. As a result, while the DFT-predicted transition occurs at in-plane lattice constants close to the experimental zero-strain lattice parameter, this lattice parameter is larger than that predicted by DFT when the unit cell is

completely optimized; the predicted transition thus occurs in the tensile region. However, the transition moves into the compressive region with slightly larger U's (see, for example, the trend above with increasing U_{Co}).

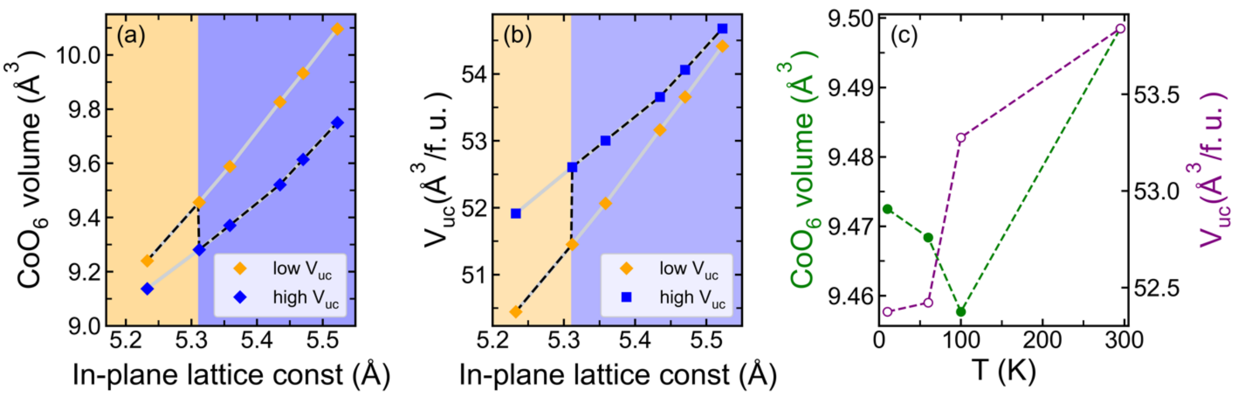

Figure S12: Supporting data for density functional theory calculations. DFT-calculated (a) CoO_6 octahedral volume and (b) unit cell volume in both the low- and high-volume phases as a function of in-plane lattice constant (and thus biaxial strain), for $U_{Co} = 3$ eV and $U_{Pr} = 4$ eV. The background color denotes which phase is energetically favorable. The gray lines highlight the trends within a given phase, while the dashed lines track the behavior of the lower-energy phase across the transition. To obtain the location of the phase boundary, we find the in-plane lattice constant for which the energy difference between the two phases is zero. This is accomplished using linear interpolation of the energy differences between neighboring data points (see the dashed line in Fig. S11(b).) In panel (c), we reproduce data from Ref. [2], showing the experimental behavior of the octahedral and unit cell volumes as a function of temperature in $Pr_{0.5}Ca_{0.5}CoO_3$ (PCCO). We find good qualitative agreement between the DFT calculations and the experimental data across the phase transition (compare (a,b) with (c)). In particular, DFT calculations predict a discontinuous drop in the unit-cell volume (and increase in the octahedral volume) across the transition with increasing compressive strain, analogous to the trends seen experimentally with decreasing temperature. This provides further evidence that the strain-driven phase transition reported in this work is similar in nature to the thermal phase transition in PCCO.

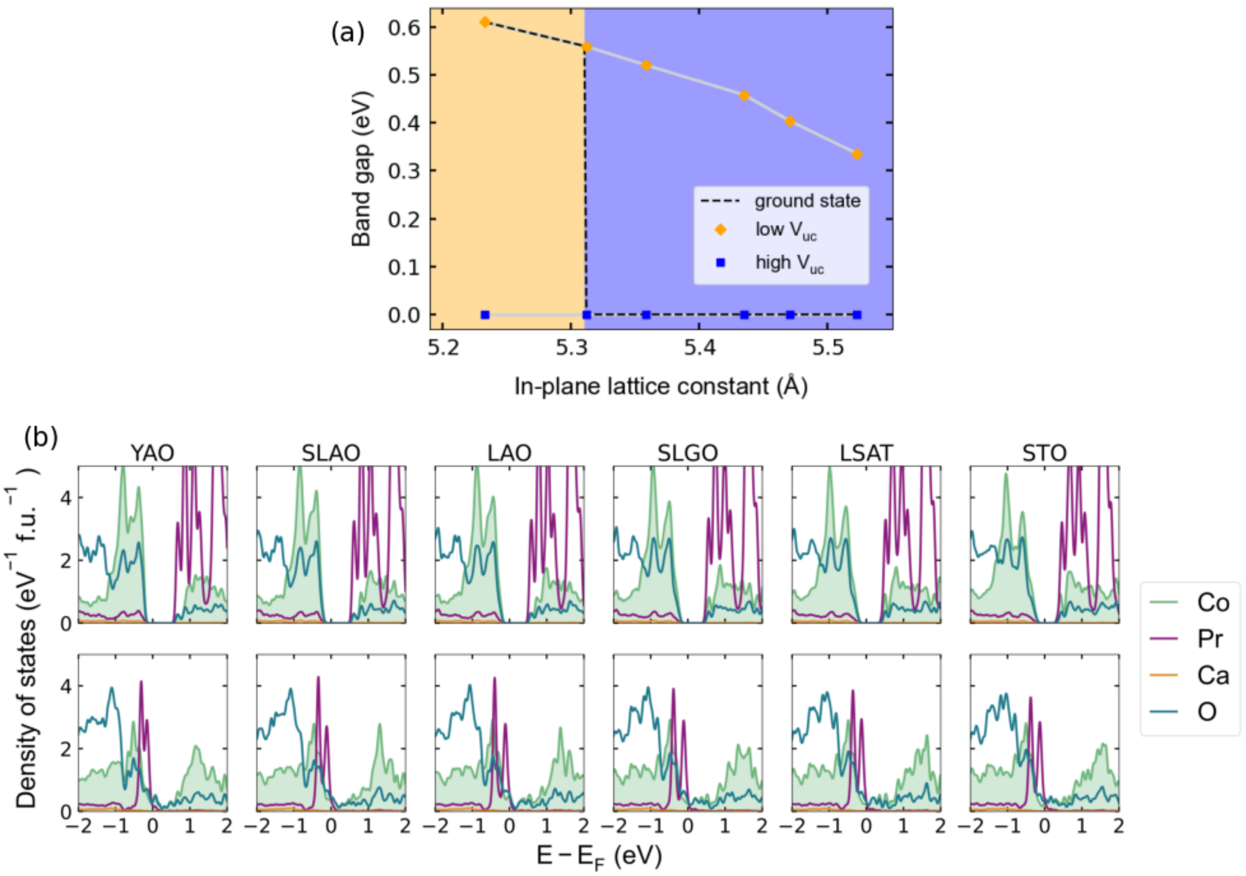

Figure S13: Supporting data for density functional theory calculations. (a) DFT-calculated band gaps as a function of in-plane lattice constant for the low- and high-volume states of $Pr_{0.5}Ca_{0.5}CoO_3$ (PCCO), for U_{Co} = 3 eV and U_{Pr} = 4 eV. The background color denotes whether the ground state is the high- or low-volume unit cell state, and the phase transition boundary is obtained where there is zero energy difference between the two phases, as described in the caption to Fig. S12. The gray lines highlight the trends within each phase, while the dashed line tracks the behavior of the band gap of the ground state across the transition. The dashed line was obtained in a similar manner to the dashed lines in Figs. S12(a,b). (b) Corresponding density-of-states (DOS) *vs*. energy plots (E_F is the Fermi energy) for each in-plane lattice constant, *i.e.*, each substrate. The top row shows the results for the low-volume unit cell states, while the bottom row is for the high-volume unit cell states. We see that the high-volume unit cell states are insulating, while the low-volume unit cell states are metallic. These calculations therefore show that there is a high-volume metallic state under tensile biaxial strain and a low-volume insulating state under compressive biaxial strain, in agreement with experiment.

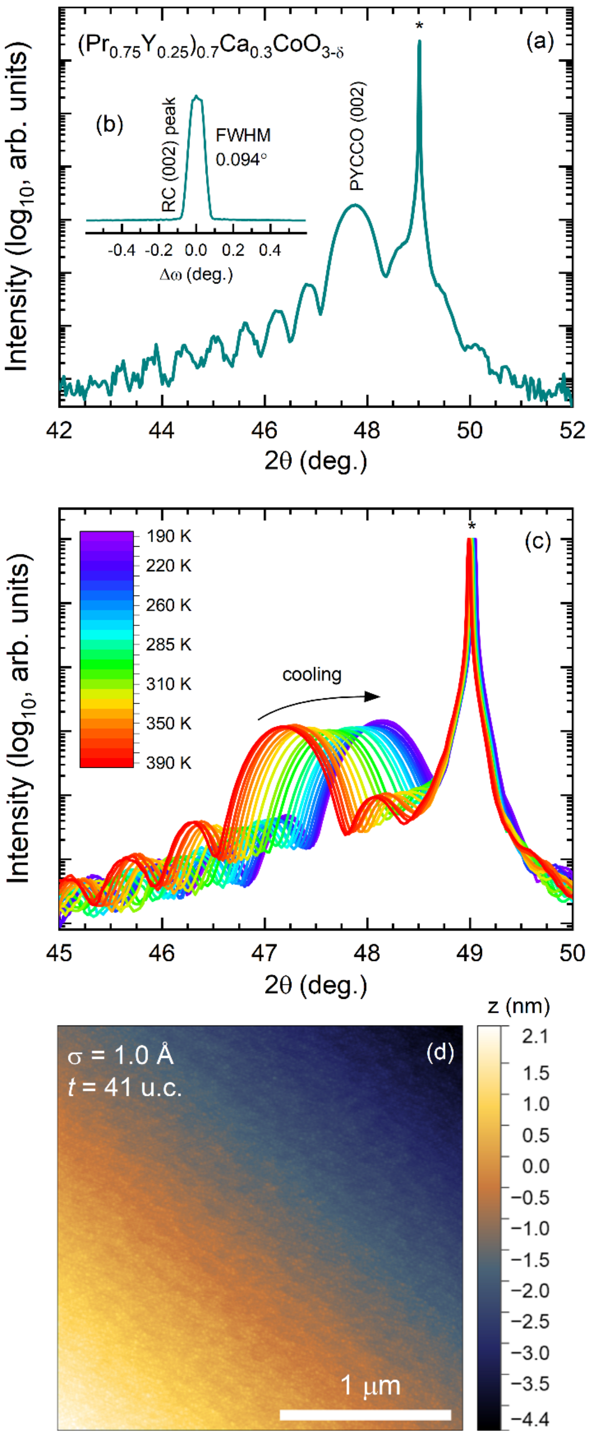

Figure S14: Structural characterization of $(Pr_{0.75}Y_{0.25})_{0.7}Ca_{0.3}CoO_{3-\delta}$ (x = 0.3, y = 0.25) films on $YAlO_3$. (a) Specular X-ray diffraction scan around the 002 film peak of the 41-unit-cell-thick $(Pr_{0.75}Y_{0.25})_{0.7}Ca_{0.3}CoO_{3-\delta}$ film on YAO(101) shown in Fig. 7(b,c). These are lab X-ray data taken using Cu K_α radiation; the substrate reflection is labelled with an "*". The extracted 300-K c-axis

lattice parameter of 3.806 Å is significantly smaller than for $(Pr_{0.85}Y_{0.15})_{0.7}Ca_{0.3}CoO_{3-\delta}$ (3.846 Å), due to the onset of the valence transition, and therefore the collapsed-volume state, even at 300 K (see Fig. 7(c)). The inset (b) shows a film 002 rocking curve (RC), with the full-width at half-maximum (FWHM) shown. (c) Temperature (T)-dependent lab X-ray diffraction around the 002 peak of the 41-unit-cell-thick $(Pr_{0.75}Y_{0.25})_{0.7}Ca_{0.3}CoO_{3-\delta}$ film on YAO(101) shown in Fig. 7(b,c), from 190 K to 390 K (see color scale). (d) Contact-mode atomic force microscopy height image of the same 41-unit-cell-thick $(Pr_{0.75}Y_{0.25})_{0.7}Ca_{0.3}CoO_{3-\delta}$ film on YAO(101), leveled to the visible terraces; the root-mean-square roughness from the displayed region is 1.0 Å.

References

1. Fitzsimmons, M. R. *et al.* Vector magnetization depth profile of a Laves-phase exchange-coupled superlattice obtained using a combined approach of micromagnetic simulation and neutron reflectometry. *Phys. Rev. B* **73**, 134413 (2006).
2. Fujita, T. *et al.* Transport and magnetic studies on the spin state transition of $Pr_{1-x}Ca_xCoO_3$ up to high pressure. *J. Phys. Soc. Jpn.* **73**, 1987–1997 (2004).
